# Molecular characterization of genome segments 1 and 3 encoding two capsid proteins of *Antheraea mylitta *cytoplasmic polyhedrosis virus

**DOI:** 10.1186/1743-422X-7-181

**Published:** 2010-08-04

**Authors:** Mrinmay Chakrabarti, Suvankar Ghorai, Saravana KK Mani, Ananta K Ghosh

**Affiliations:** 1 Department of Biotechnology, Indian Institute of Technology Kharagpur, Kharagpur 721302, West Bengal, India

## Abstract

**Background:**

*Antheraea mylitta *cytoplasmic polyhedrosis virus (AmCPV), a cypovirus of *Reoviridae *family, infects Indian non-mulberry silkworm, *Antheraea mylitta*, and contains 11 segmented double stranded RNA (S1-S11) in its genome. Some of its genome segments (S2 and S6-S11) have been previously characterized but genome segments encoding viral capsid have not been characterized.

**Results:**

In this study genome segments 1 (S1) and 3 (S3) of AmCPV were converted to cDNA, cloned and sequenced. S1 consisted of 3852 nucleotides, with one long ORF of 3735 nucleotides and could encode a protein of 1245 amino acids with molecular mass of ~141 kDa. Similarly, S3 consisted of 3784 nucleotides having a long ORF of 3630 nucleotides and could encode a protein of 1210 amino acids with molecular mass of ~137 kDa. BLAST analysis showed 20-22% homology of S1 and S3 sequence with spike and capsid proteins, respectively, of other closely related *cypoviruses *like *Bombyx mori *CPV (BmCPV), *Lymantria dispar *CPV (LdCPV), and *Dendrolimus punctatus *CPV (DpCPV). The ORFs of S1 and S3 were expressed as 141 kDa and 137 kDa insoluble His-tagged fusion proteins, respectively, in *Escherichia coli *M15 cells via pQE-30 vector, purified through Ni-NTA chromatography and polyclonal antibodies were raised. Immunoblot analysis of purified polyhedra, virion particles and virus infected mid-gut cells with the raised anti-p137 and anti-p141 antibodies showed specific immunoreactive bands and suggest that S1 and S3 may code for viral structural proteins. Expression of S1 and S3 ORFs in insect cells via baculovirus recombinants showed to produce viral like particles (VLPs) by transmission electron microscopy. Immunogold staining showed that S3 encoded proteins self assembled to form viral outer capsid and VLPs maintained their stability at different pH in presence of S1 encoded protein.

**Conclusion:**

Our results of cloning, sequencing and functional analysis of AmCPV S1 and S3 indicate that S3 encoded viral structural proteins can self assemble to form viral outer capsid and S1 encoded protein remains associated with it as inner capsid to maintain the stability. Further studies will help to understand the molecular mechanism of capsid formation during cypovirus replication.

## Background

Cytoplasmic polyhedrosis virus or CPV of the genus *Cypovirus *of *Reoviridae *family [1,2] infects the midgut of the wide range of insects belonging to the order Diptera, Hymenoptera and Lepidoptera [3,4]. Like other members of *Reoviridae*, CPV genome is also composed of 10 double stranded RNA segments (dsRNA) (S1-S10) [2]. A small eleventh segment (S11) has been reported in some cases such as *Bombyx mori *CPV (BmCPV) [5] and *Trychoplusia ni *CPV (TnCPV) [6]. Each dsRNA segment is composed of a plus mRNA strand and it's complementary minus strand in an end to end base pair configuration except for a protruding 5' cap on the plus strand. On the basis of electrophoretic migration patterns of the dsRNA segments in agarose or acrylamide gels, CPVs have been classified into 16 different types [1,7]. CPVs are self competent for transcription, possessing all the enzymes necessary for mRNA synthesis and processing [8]. BmCPV, the type Cypovirus, has a single layer capsid made up of 120 copies of the major capsid protein, VP1, which is decorated with 12 turrets on its icosahedral vertices [9,10]. These hollow turrets are involved in post-transcriptional processing of viral mRNA and provide a channel through which newly synthesized 5'capped viral RNA are released from the capsid into the cytoplasm of infected cells [10,11]. After translation of this mRNA into capsid, polymerase and other proteins, they assembled into viral procapsid within which one copy of each genome segments plus polarity RNA are packaged and replicated to form dsRNA. CPV capsids thus formed can be released as non-occluded virus particles to directly infect fresh neighboring cells or occluded in a viral protein matrix called polyhedrin to form polyhedra [12]. It has been reported that VP1 protein, encoded by genome segment 1 of BmCPV, can self assemble to form single shelled virus like particles (VLPs) [13,14] and their stability is maintained by interaction with VP3 and VP4 proteins encoded by genome segments 3 and 4, respectively [15,16]. Recent cryo-electron microscopic study has shown the region of capsid protein directly interacting with viral RNA indicating the role of capsid in RNA packaging, replication and transcription [17]. Therefore, understanding the assembly of capsid not only provides insight into in the virus life cycle but also helps to develop mechanism for the disruption of virus assembly for therapeutic application [18]. But besides BmCPV, capsids of other CPVs have not been studied well although all the genome segments of DpCPV, LdCPV and TnCPV have been cloned and sequenced [6,19-21].

*Antheraea mylitta *cytoplasmic polyhedrosis virus (AmCPV) is one of the most widespread pathogens of Indian non-mulberry silkworm, *A. mylitta*. CPV-infected *A. myllita *larvae develop chronic diarrhea that eventually leads to a condition known as "Grasserie" and ultimate death [22]. Almost 20-30% larval mortality occurs annually due to this virus attack [22]. We have previously characterized the structure of AmCPV by electron microscopy and its genome by electrophoresis which reveals that it is similar to that of a type- 4 CPV and consists of 11 ds RNA molecules [23]. We have also reported that the genome segments 6, 7, 8 of AmCPV encode viral structural proteins [24-26], segment 2 encodes viral RNA dependent RNA polymerase [27], segment 9 encodes a nonstructural protein, NSP38, having RNA binding property [28], segment 10 codes for polyhedrin [29] and segment 11 does not code for any protein [26]. But the genome segments encoding viral capsid proteins have not been characterized. Here, we report molecular cloning, sequencing and expression of S1 and S3 of AmCPV in *E. Coli *via bacterial expression vector as well as in insect cells using a baculovirus system and show by functional analysis that S3 encoded protein can self assemble into capsid and S1 encoded protein remains associated with the capsid to maintain its stability.

## Results and discussion

### Genetic analysis of AmCPV S1 and S3

AmCPV S1 and S3 RNA were isolated, converted to cDNA and cloned into pCR-XL-TOPO and the total nucleotide sequences were determined in both forward and reverse directions. S1 consisted of 3852 nucleotides with one long ORF of 3735 nucleotides and could encode a protein of 1245 amino acids with molecular mass of ~141 kDa (p141). Thirty four nucleotides upstream of translation initiation codon (ATG) and 80 nucleotides downstream of translation stop codon (TGA) were present at untranslated sequences (Genbank accession No: HM230690). Similarly, S3 consisted of 3784 nucleotides having a long ORF of 3630 nucleotides and could encode a protein of 1210 amino acids with molecular mass of ~137 kDa (p137). Twenty seven nucleotides upstream of translation initiation codon (ATG) and 124 nucleotides downstream of translation stop codon (TGA) were present as untranslated sequences (Genbank accession No: HM230691). Cloning of S1 and S3 was confirmed by northern analysis of total AmCPV RNA using cloned S1 and S3 cDNA as probes (data not shown).

BLASTP results showed 22%, 23% and 27% homology of AmCPVS1 encoded p141 with segment 3 encoded proteins VP3, VP2 and a hypothetical protein of BmCPV1, DpCPV1 and LdCPV14, respectively [13,20,21]. Function of VP3 protein of BmCPV1 is not exactly known but probably codes for spike protein [13]. Therefore it is suggested that AmCPV S1 may also code for a minor capsid protein which is probably involved in spike formation. AmCPV S1 contained seventeen N-linked glycosylation sites, two cAMP- and cGMP-dependent protein kinase phosphorylation sites, twenty casein kinase II phosphorylation sites, twelve N-myristoylation sites, fourteen protein kinase C phosphorylation sites and two tyrosine kinase phosphorylation sites. Secondary structure prediction with PHD and GOR4 showed that 36.54% of the residues are likely to form α-helices, 25.69% would form extended sheets and 37.77% would form random coils. But their functional significance remains to be determined.

BLASTP results also showed 20-23% homology of AmCPV S3 encoded p137 with segment 1 encoded major capsid protein, VP1, of BmCPV, DpCPV and LdCPV indicating that AmCPVS3 may code for major capsid protein of AmCPV. AmCPV S3 contained eight N-linked glycosylation sites, one cAMP- and cGMP-dependent protein kinase phosphorylation site, 14 protein kinase C phosphorylation sites, 19 casein kinase II phosphorylation sites, 13 N-myristoylation sites and one prokaryotic membrane lipoprotein lipid attachment site. Secondary structure prediction with PHD and GOR4 showed that 28% of the residues are likely to form α-helices, 14.9% would form extended sheets and 57.1% would form random coils. But the correlation between this structure and function remains to be made. In both the genes the 5' terminal sequence AGTAAT and 3' terminal sequence AGAGC were found to be the same as the 5' and 3' terminal sequences found in AmCPV genome segments 2, 6, 7, 8 and 10 indicating that the genome structure of this CPV may follow the same pattern as found in other CPVs [6,19-21,30].

**Figure 1:**
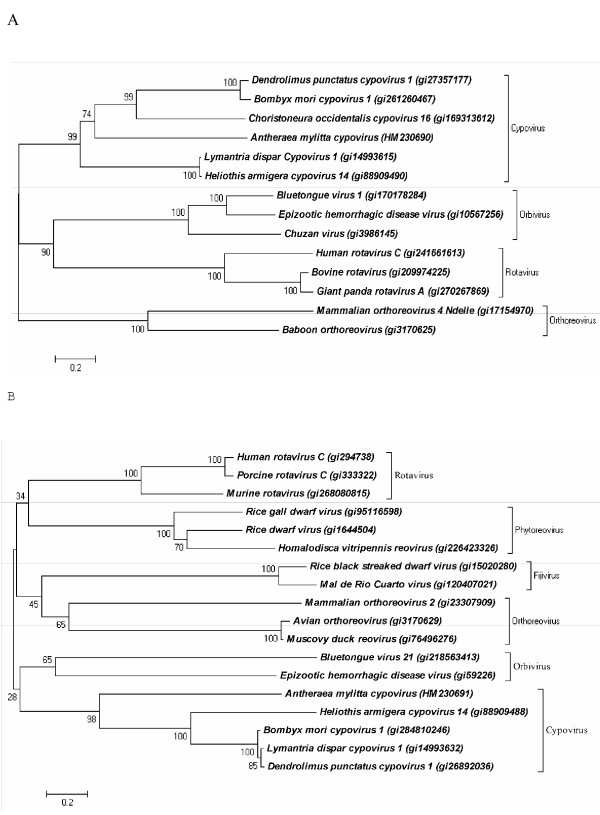
**Phylogenetic analysis of AmCPV S1 (A) and AmCPV S3 (B) encoded proteins with other members of the *Reoviridae***. The number at each node represents bootstrap value of 100 replicates. GenBank accession numbers are shown in parenthesis.

Phylogenetic analysis of AmCPV S1 and S3 amino acid sequences with other viruses in the *Reoviridae *showed its close relatedness with some members of the cypovirus such as BmCPV-1, DpCPV and LdCPV (Fig. 1A &1B) and indicates that all these *cypoviruses *may have been originated from a common ancestral insect virus.

### Analysis of recombinant AmCPV S1 and S3 encoded proteins expressed in *E. coli *and insect cells

**Figure 2:**
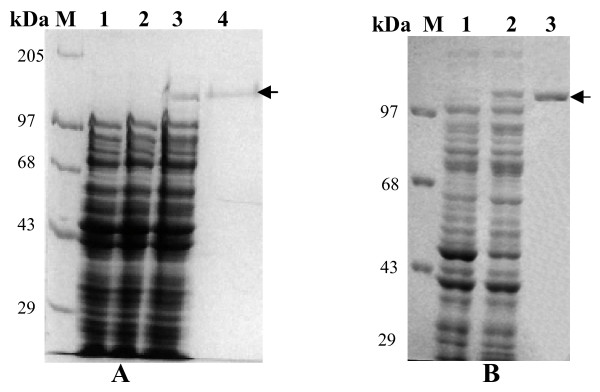
**(A) Analysis of *E. coli *M15 expressed AmCPV S1 encoded protein by SDS-8% PAGE**. Lane M, Molecular weight marker (Bangalore Genei); lane 1, uninduced cell lysate; lane 2, induced cell supernatant; lane 3, induced cell pellet; lane 4, Ni-NTA purified protein. **(B) Analysis of *E. coli *M15 expressed AmCPV S3 encoded protein by SDS-8% PAGE**. Lane M, Molecular weight marker (Bangalore genei); lane 1, uninduced cell lysate; lane 2, induced cell lyaste; lane 3, Ni-NTA purified protein.

AmCPV S1 and S3 were expressed in *E. coli *M15 cells as insoluble 141 kDa (Fig. 2A, lanes 3 & 4) and 137 kDa (Fig. 2B, lanes 2 & 3) proteins, respectively. Polyclonal antibodies were raised in a rabbit against purified p141 and p137, and titered as 10^-4 ^by ELISA.

**Figure 3:**
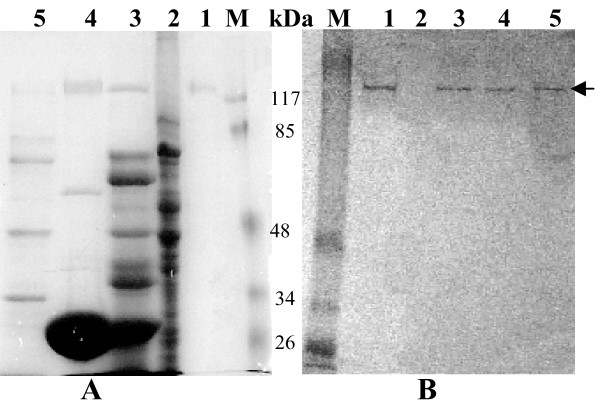
**Immunoblot analysis of AmCPV S1 encoded proteins using anti-p141 polyclonal antibody**. (A) SDS-8% PAGE and (B) Western Blot. Lane M, Prestained protein molecular weight marker (Fermentas); lane 1, purified insect cell expressed recombinant p141 protein; lane 2, uninfected midgut of *A. mylitta*; lane 3, infected midgut of *A. mylitta*; lane 4, purified polyhedra and lane 5, purified virion particle. Arrow indicates the position of immunoreactive protein.

**Figure 4:**
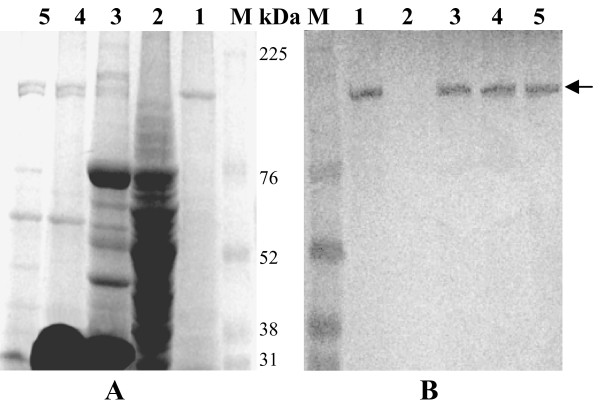
**Immunoblot analysis of AmCPV S3 encoded proteins using anti-p137 polyclonal antibody**. (A) SDS-8% PAGE and (B) Western Blot. Lane M, Prestained molecular weight marker (GE); lane 1, purified insect cell expressed recombinant p137 protein; lane 2, uninfected midgut of *A. mylitta*; lane 3, infected midgut of *A. mylitta; *lane 4, purified polyhedra; and lane 5, purified virion particle. Arrow indicates the position of immunoreactive protein

Sf9 cells infected with S1 and S3 recombinant baculovirus expressed these proteins in soluble form as 141 and 137 kDa, respectively [Fig 3A and 4A (lane 1)]. This was confirmed by immunoblot analysis (Fig. 3B and 4B, lane 1). Expression of predicted same size proteins both in bacteria and insect cells indicate that although a number of glycosylation sites are present in both these genes but they are not used for post translational modification. In *E. coli *M15 cells the expressed proteins might not fold properly into correct conformation and thus the incorrectly folded protein may have aggregated to produce insoluble inclusion bodies but in insect (Sf9) cells via baculovirus expression system due to proper folding soluble proteins are produced.

To determine function of AmCPV S1 and S3 encoded proteins, immunoblot analysis was done with the midgut of uninfected and virus-infected larvae, polyhedra and virion particles using purified polyclonal anti-p141 and anti-p137 antibodies. Major immunoreactive bands of 141 kDa and 137 kDa (Fig. 3 &4, lanes 3, 4 and 5) were observed in infected midgut, purified polyhedra as well as virus particles, but not in uninfected midgut (lane 2) indicating that they might code for two viral structural proteins.

### Transmission Electron Microscopic (TEM) analysis of virus like particles

**Figure 5:**
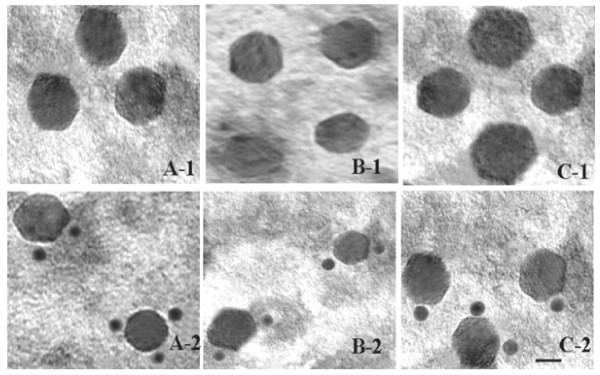
**Electron micrographs of uranyl acetate-stained native and recombinant VLPs of AmCPV**. (A) Native AmCPV particles; (B) Recombinant VLPs expressing AmCPV S3 encoded protein; (C) Recombinant VLPs expressing AmCPV S1 and AmCPV S3 encoded proteins. Upper panel (A-1, B-1 and C-1) shows the purified particles in 20 mM PBS, pH 7.3 and lower panel (A-2, B-2, and C-2) shows immunogold staining of these particles. Bar, 20 nm.

To visualize the formation of virus like particles (VLPs) in recombinant baculovirus infected Sf9 cells and to confirm the identity of their protein content, VLPs were purified from infected cells and immunogold staining of the particles were performed using rabbit anti-p141 or anti-p137 antibodies. As shown by TEM analysis (Fig. 5. A-2, B-2, C-2), native AmCPV, recombinant VLP from Sf 9 cells infected with AmCPV S3 baculovirus recombinants alone or, Sf9 cells co-infected with AmCPV S1 and S3 baculovirus recombinants were specifically labeled with rabbit anti-p137 antibody conjugated gold particles. No gold particle labeling was observed when anti-p141 antibody was used (data not shown). Also no VLP formation was observed in cells infected with AmCPVS1 recombinant baculovirus alone. These results indicate that AmCPV S3 encoded protein alone has the ability to self assemble for the formation of single shelled particle (capsid) without the assistance of any other structural protein of AmCPV. Similar capsid formation has been reported for BmCPV S1 encoded VP1 protein [14]. No gold particle labeling in VLPs produced from Sf9 cells co-infected with AmCPV S1 and S3 recombinants using anti-p141 antibody may be due to presence of S1 encoded protein in the inner side of capsid where antibody can not access or absence of epitope (exposed outside) specific antibody in the raised polyclonal antibody.

### Immunoblot analysis of VLPs

**Figure 6:**
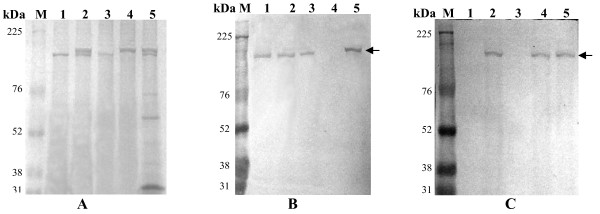
**Immunoblot analysis of recombinant VLPs using anti-p137 and anti-p141 antibodies**. (A) SDS-8% PAGE, (B) western blot with anti-p137 antibody and (C) western blot with anti-p141 antibody. Lane M, Prestained molecular weight marker (GE Healthcare Bio-Science); lane 1, VLPs from cells infected with AmCPVS1 recombinant baculovirus, lane 2, VLPs from cells infected with AmCPVS1 and AmCPV S3 recombinant baculovirus; lane 3, purified p137 protein; lane 4, purified p141 protein; lane 5, purified native virion particles. Arrow indicates the position of immunoreactive protein.

To further confirm the protein content of these VLPs obtained from recombinant baculovirus infected Sf9 cells, immunoblot analysis was performed using anti-p137 and anti-p141 antibodies. Immunoblot study using anti-p137 antibody (Fig. 6B) showed a single major immunoreactive band at 137 kDa in purified VLPs from cells infected with AmCPV S3 baculovirus recombinants (lane 1), purified VLPs from cells co-infected with both AmCPV S1 and S3 baculovirus recombinants (lane 2), purified p137 protein (lane 3) and native virion particles (lane 5). Similar immunoblot study, using anti-p141 antibody showed a single major immunoreactive band at 141 kDa in purified VLPs obtained from cells expressing both AmCPV S1 and S3 (Fig. 6C, lane 2), purified recombinant p141 protein (lane 4) and native virion particles (lane 5). Since in SDS-PAGE, after Coomassie blue staining two bands (137 kDa and 141 kDa) were observed in purified VLPs from cells co-infected with both AmCPV S1 and S3 baculovirus recombinants (lane 2), and also in purified native virion particles (lane 5) and reacted with both anti-p141 and p137 antibodies, these results indicate that p137 is involved in the formation capsid outer shell and p141 is associated in the inner side of capsid (VLPs). Three dimensional structure of BmCPV has shown presence of spike molecules and transcription enzyme complexes along the icosahedral five fold axis both inside and outside of the core particles [10,16]. Similar studies are required to understand the association of AmCPV S1 and S3 encoded proteins in the viral capsid.

### Comparison of stability of native virion and virus like particles at different pH

**Figure 7:**
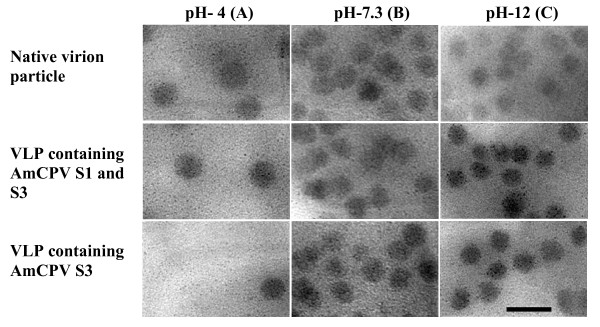
**Transmission electron micrographs (TEM) of uranyl acetate-stained native virions and purified VLPs after incubation at different pH**. Native virions, purified VLPs containing both AmCPV S1 and S3 encoded proteins, or AmCPV S3 encoded protein alone was incubated at pH-4 (A), pH-7.3 (B), and at pH-12 (C) and analyzed by TEM at 50 kV. Bar 100 nm.

Transmission electron microscopic studies of native virions and recombinant VLPs at different pH showed that VLPs are more stable in alkaline condition rather than acidic pH (Fig. 7). Most of VLPs maintained their intact structure at pH-12 whereas totally disintegrated at below pH-4. At any given pH native virion particles were found to be more stable than VLPs made up of p137 or p137 and p141 together (Table 1). But VLPs composed of both p137 and p141 were found more stable than VLPs made up of p137 alone. These results again confirmed that AmCPV S1 encoded 137 kDa protein forms the major outer capsid protein and S3 encoded 141 kDa protein remains associated with it and plays an important role in maturation of virus particles by maintaining the stability and integrity of the capsid protein. Since the stability of native virion is more than recombinant VLPs it is suggested that similar to BmCPV [14] in addition to S1 and S3 encoded protein other virion proteins encoded by other genome segments may also helps in maintaining the stability and integrity of capsid. Characterization of all the AmCPV genome segments will help to elucidate the role of other proteins which may be involved in capsid formation.

**Table 1:** Stability of native virions and virus like particles at different pH.

Sample	20 mM PBS (pH-12)	20 mM PBS (pH-9)	20 mM PBS (pH-7.3)	**0.2 M NaH**_**2**_**PO**_**4 **_**(pH-5)**	**0.2 M NaH**_**2**_**PO**_**4 **_**(pH-4)**	**0.2 M NaH**_**2**_**PO**_**4 **_**(pH-3)**
**Native virion**	87%	90%	100%	83%	19%	0%

**VLPs containing AmCPV S3 encoded protein**	80%	83%	100%	69%	11%	0%

**VLPs containing AmCPV S1 & S3 encoded protein**	81%	89%	100%	81%	16%	0%

Values are average of three assays.

## Conclusions

AmCPV genome segments 1 and 3 have been cloned and expressed in insect cells via baculovirus recombinants. Analysis of expressed protein produced in insect cells by TEM, immunogold and immunoblot analysis indicates that AmCPV S3 encodes major outer capsid protein which can self assemble into VLPs whereas AmCPV S1 codes for an inner minor capsid protein which may be involved in stabilizing virion structure. These studies of capsid assembly and formation will help to understand viral life cycles and to develop mechanism which can disrupt virus assembly for therapeutic application.

## Methods

### Silkworm, Virus, Cell lines

The CPV infected Indian non-mulberry silkworms, *A. mylitta*, were collected from different tasar farms of West Bengal and Jharkhand states of India. The *Spodoptera frugiperda *cell line, Sf9, was procured from Invitrogen, USA and maintained on TNM-FH (Grace Insect media) supplemented with 10% fetal bovine serum (Hyclone) and lactalbumin hydrolysate and yeastolate (Invitrogen) at 27°C.

### Purification of polyhedral bodies, isolation of total genomic RNA and extraction of genome segment S1 and S3 RNA

Polyhedra were purified from mid guts of infected silkworm larvae by sucrose density gradient centrifugation according to the method of Hayashi and Bird [31] with some modification [23]. Genomic RNA was extracted from the purified polyhedra by the standard guanidinium isothiocyanate method [32] and fractionated in 6% PAGE. Genome segments 1 and 3 were precisely excised from gels and were eluted by the crush and soak method [33].

### Molecular cloning and sequencing of genome segment S1 and S3

S1 and S3 genomic RNA of AmCPV were converted to cDNA as described by a sequence independent RT method [34] using two primers (AG1 and AG2). The 3'-end of 5'-phosphorylated primer, AG1 (Table 2), was blocked by NH_2 _to prevent its concatenation in subsequent dsRNA/DNA ligation reactions. Approximately, 200 ng of purified S1 and S3 RNA segments were taken and in each case primer AG1 was ligated to both 3' ends of dsRNA by using T4 RNA ligase for 1 hour at 37°C. The tailed RNA was denatured by heating, annealed to primer AG2 (Table 2), which is complementary to AG1, and reverse transcribed at 55°C for 50 min by using Thermoscript reverse transcriptase (Invitrogen). The template RNA from RNA/cDNA hybrid was removed by digestion with RNaseH and cDNA strands of both polarities were annealed by incubating at 65°C for 2 h. The cDNA ends were repaired by incubation with *Taq *DNA polymerase (Bioline) at 72°C for 20 min and cloned into pCR-XL-TOPO vector (Invitrogen) to make plasmid pCR-XL-TOPO/AmCPVS1 and pCR-XL-TOPO/AmCPVS3. After transforming in *E. coli *TOP 10 cells, plasmids were isolated and characterized by *Eco*RI digestion. Recombinant plasmids containing proper size insert were then sequenced by using Bigdye terminator in an automated DNA sequencer (ABI, model 3100) with M13 forward and reverse primers as well as internal primers designed from deduced sequences.

**Table 2:** List of primers used for cDNA synthesis and cloning of AmCPV S1 and S3.

Sl. No	Primer name	Primer sequence	Restriction Sites (Bold)
1	AGCPV145F	5'-TCTTGCGGC**GAGCTC**ACGTCAATG-3'	*Sac*I
2	AGCPV146R	5'-TGTATATGAA**GTCGAC**TCTATTATCAG-3'	*Sal*I
3	AGCPV154F	5'-CGCCCT**GGATCC**AGAATGGAG-3'	*Bam*HI
4	AGCPV157R	5'-CCTACTATC**AAGCTT**CGAATG-3'	*Hin*dIII
5	AG1	5' PO_4 _-CCCGGATCCGTCGACGAATTCTTT-NH_2_-3'	
6	AG2	5'-AAAGAATTCGTCGACGGATCCGGG-3'	

### Sequence analysis

Genome sequence of AmCPVS1 and S3 were analyzed by Sequencher program (Gene codes corporation, USA) and homology search was done using BLAST [35]. Conserved motifs were identified using motif scan program (http://myhits.isb-sib.ch/egi-bin/motif_scan). The molecular weight of deduced protein, and amino acid compositions were determined using protein calculator program (http://www.scripps.edu/~edputnam/protealc.html). Secondary structure was predicted using PHD and GOR4 programs [36]. To understand the evolutionary relationship between AmCPV and other members of *Reoviridae*, the amino acid sequences of AmCPVS1 and S3 were compared with those of other reoviruses and cypoviruses, and Phylogenetic trees were generated by neighbor-joining method with the program MEGA (http://www.megasoftware.net/index.html) [37]. Tree drawing was performed with the aid of TREEVIEW program [38].

### Expression and purification of AmCPV genome S1 and S3 encoded protein from *E. coli*

The entire protein coding regions of AmCPVS1 (from nucleotide 35 to 3769) and S3 (from nucleotide 28 to 3657) cDNA were amplified by PCR from pCR-XL-TOPO/AmCPVS1 and pCR-XL-TOPO/AmCPVS3 by *accuzyme *DNA polymerase (Bioline) and two sets of synthetic primers, AGCPV 154F and AGCPV 157R, and AGCPV145F and AGCPV 146R (Table 2), respectively, and analyzed by 1% agarose gel electrophoresis. The amplified PCR products were restriction digested and cloned into pQE-30 vector. The resulting recombinant plasmids, pQE-30/AmCPVS1 and pQE-30/AmCPVS3, were then transformed into *E. coli *M15 cells and the colonies were screened following restriction digestion. For protein expression, the recombinant M15 *E. coli *cells were grown in 5 ml LB medium containing 100 μg/ml of ampicillin and 25 μg/ml of kanamycin until the O.D (at 600 nm) reached to 0.6 at 37°C and induced with 1 mM IPTG for another 5 hours at the same temperature. Bacteria were then harvested by centrifugation, lysed by boiling with sample loading buffer (60 mM Tris-HCl, pH 6.8, 10% Glycerol, 2% SDS, 5% β-mercaptoethanol and 1 μg/ml bromophenol blue) for 3 min. and analyzed by SDS-8% PAGE [39]. The molecular mass of the encoded protein was determined by comparison with standard protein molecular weight markers using Kodak software 1D, version 3.6.3.

For large scale protein expression recombinant *E. coli *M15 containing pQE-30/AmCPVS1 and pQE-30/AmCPVS3 were grown in 1 L LB medium till OD at 600nm reached to 6.0 and then induced with 1 mM IPTG for 4 hour. After harvesting bacteria by centrifugation, the insoluble 6× His-tagged AmCPV S1 and S3 encoded fusion proteins were purified from the bacterial lysate by Ni-NTA affinity chromatography according to the manufacturer's protocol (Qiagen) and eluted from the Ni-NTA column by buffer containing 250 mM imidazole [24,25]. The amount of the purified protein was determined by the method of Bradford [40] using BSA as standard and the purity was checked by SDS-8% PAGE.

### Rabbit immunization and production of polyclonal antibodies

The Ni-NTA purified S1 and S3 proteins were concentrated using centricon (Millipore) according to the manufacturer's protocol and analyzed by SDS-PAGE. After electro elution of protein bands from SDS-PAGE, approximately 600 μg of protein was mixed with Freund's complete adjuvant and injected subcutaneously at multiple sites in a rabbit [28,41]. Four more booster doses were given with Freund's incomplete adjuvant with the same amount of protein via the same route at 4-week interval. Blood was collected 10 days after the final booster, serum prepared and the antibody titer was determined by ELISA [41].

### Construction of recombinant baculovirus and expression of AmCPVS1 and S3 in Sf9 cells

The entire protein coding regions of AmCPVS1 and AmCPVS3 cDNA were amplified by PCR from pCR-XL-TOPO/AmCPV S1 and pCR-XL-TOPO/AmCPVS3 by *accuzyme *DNA polymerase (Bioline) using two sets of synthetic primers, AGCPV 154F and AGCPV 157R, and AGCPV 145F and AGCPV 146R, respectively, and were analyzed by 1% agarose gel electrophoresis. The PCR amplified products were eluted from the gel after restriction digestion and cloned into pBluebacHis2A baculovirus transfer vector (Invitrogen) upstream of baculovirus polyhedrin promoter. The resulting recombinant baculovirus transfer vectors and *Bsu*I digested linearized *Autographa californica *nucleopolyhedrosis virus or AcMNPV DNA were co-transfected into Sf9 cells using insectin plus according to the manufacturer's protocol (Invitrogen). Briefly, log phase grown Sf9 cells (10^6^) were seeded in each Petri dish and allowed to adhere for 1 h before transfection and were washed twice with serum free medium. These cells were then co-transfected with 4 μg of pBluebacHis2A/AmCPV S1 or pBluebacHis2A/AmCPV S3 plasmids mixed with 0.5 μg of linearized Bac-N-Blue DNA (Invitrogen) using the supplied liposome. The transfected cells were incubated for 72 h at 27°C and culture medium was collected. After infecting fresh Sf9 cells with this culture supernatant, recombinant baculovirus (blue plaques) were isolated by plaque purification [27]. To produce recombinant AmCPVS1 or S3 encoded proteins, Sf9 cells were cultured in 1-L spinner flask (2 × 10^7 ^cells) and infected with recombinant baculovirus at an m.o.i of five. The cells were harvested 72 h post-infection and washed twice with phosphate buffer saline (137 mM NaCl, 10 mM phosphate, 2.7 mM HCl, pH 7.3). The washed cell pellet was resuspended in ice-cold lysis buffer (20 mM Tris-Cl, [pH-7.5], 1.0 mM EDTA, 10 mM dithiothreitol [DTT], 2% Triton X-100, 500 mM NaCl, 50% glycerol) containing protease inhibitor cocktail (Sigma), lysed by sonication, centrifuged at 3000 g for 30 min at 4°C to clear the debris and the supernatant was used to purify proteins by Ni-NTA affinity chromatography. In brief, the supernatant was incubated for 1 h on ice with Ni-NTA sepharose (Qiagen) pre-equilibrated with the lysis buffer. After washing unbound proteins with 10-column volume of lysis buffer, bound AmCPVS1 or S3 encoded proteins were eluted from the beads with elution buffer (10 mM Tris-HCl, 50 mM NaCl, 250 mM imidazole, pH-7.5), concentrated by Centricon (Millipore) and analyzed by SDS-8% PAGE.

### Immunoblot analysis of S1 ad S3 encoded proteins

Detection of AmCPVS1 and S3 encoded protein in infected cells was done by western blot analysis using polyclonal antibodies raised against bacterially-expressed p141 or p137 proteins in rabbit. Ni-NTA purified AmCPVS1 and S3 encoded protein from baculovirus infected insect cells, the midgut of uninfected and AmCPV-infected fifth instar larvae, purified polyhedra, and purified virions were resolved by SDS-8% PAGE. Following electrophoresis, proteins from the gel were transferred onto nitrocellulose membranes (Stratagene). After blocking with 3% BSA, the membranes were washed with 1× PBS and incubated with 200 times diluted affinity purified anti-p141 or anti-p137 polyclonal antibodies for 1 h at 20-25°C. After washing with 1× PBS as above, the membrane was incubated with protein A-conjugated horseradish peroxidase at a dilution of 1:5000 for 1 h, washed three times with 1× PBS and color development was done using the HPO color development kit (Bio-Rad).

### Isolation of native virus from polyhedral bodies, and virus like particles from recombinant baculovirus infected Sf 9 cells

Native virus particles were isolated from polyhedral bodies according to the method Hayashi and Bird [31] with some modification [23]. In brief, sucrose density gradient purified polyhedral bodies were lysed by 0.2 M carbonate buffer (pH 10.2) and neutralized by 0.2N HCl. After separating the intact polyhedral bodies by centrifugation at 30,000 g for 5 min, the virus particles were pelleted by centrifugation at 94,500 g for 90 min at 4°C and finally resuspended in 20 mM PBS, pH-7.3.

Sf 9 cells infected with recombinant baculoviruses (expressing either S1 or S3 alone or S1 and S3 together) were harvested by centrifugation at 1200 rpm for 10 min after 72 h post infection incubation. After three washes with PBS, cells were resuspended in lysis buffer (10 mM Tris-HCl, 0.15 M NaCl, 5 mM MgCl_2 _, pH7.4), sonicated and supernatant was collected after centrifugation at 30,000 g for 5 min. The supernatant was subjected to a 10-40% sucrose density gradient centrifugation at 94,500 g for 90 min. The band materials were collected, diluted with PBS and VLPs were pelleted by centrifugation at 1,50,000 g for 90 min. The pellet was washed, resuspended in 20 mM PBS, pH-7.3 and observed by TEM [42].

### Immunogold labeling and analysis of virus like particles by transmission electron microscopy

To confirm the formation of virus like particles by AmCPV S3 and S1 encoded proteins, immunogold staining of the particles was performed according to the method described by Lin [43]. Briefly, after absorption of virus particles on the carbon coated grids, blocking was done using 1% BSA in 20 mM PBS. After washing with 20 mM PBS, affinity purified anti-p137 polyclonal antibody or anti-p141 polyclonal antibody raised in rabbit was added at a dilution of 1:100, and incubated for 30 min. Then carbon coated grids were washed again with 20 mM PBS and gold tag anti-rabbit IgG was added at a dilution of 1:100. The grids were then washed three times with water and the samples were stained with 2% aqueous uranyl acetate. A set of controls without gold particle, was also done for the native virion and recombinant virus like particles (VLPs). After overnight vacuum drying, samples were examined in a JEM-2100 HRTEM operating at 200 kV.

### Immunoblot analysis of virion like particles

For detecting the presence of AmCPVS1 and S3 encoded proteins in the VLPs produced in insect cells infected with recombinant baculovirus expressing either S3 alone, or S1 and S3 together, and in native virions purified from polyhedra, western blot analysis was done using anti-p137 and anti-p141 antibodies. In brief, purified virus like particles, Ni-NTA purified protein samples from recombinant baculovirus infected Sf9 cells and native virions were resolved by SDS-8% PAGE and the gel was transferred onto nitrocellulose membranes (Stratagene). Western blot study was performed following the same protocol as described above using 1:200 fold diluted anti-p137 and anti-p141 antibody.

### Stability of native virus and recombinant virus like particles at different pH

To compare the stabilities of VLPs produced in insect cells infected with recombinant baculovirus expressing either S3 alone, or S1 and S3 together, with respect to native virions, VLPs and density gradient purified native viral particles were resuspended in 20 mM PBS or 0.2 M NaH_2_PO_4 _of different pH ranging from 3 to 12 and incubated at room temperature for 10 min. After incubation they were observed in transmission electron microscopy operating at 50 kV in different microscopic fields.

## Competing interests

The authors declare that they have no competing interests.

## Authors' contributions

MC and SKKM designed the research study, performed experiments and contributed to the writing of manuscript. SG helped in analyzing the data. AKG supervised the work and contributed to the writing of the manuscript. All authors read and approved the final version of the manuscript.
