# Editorial Expression of Concern: Molecular characterization of genome segments 1 and 3 encoding two capsid proteins of *Antheraea mylitta* cytoplasmic polyhedrosis virus

**DOI:** 10.1186/s12985-021-01648-3

**Published:** 2021-08-31

**Authors:** Mrinmay Chakrabarti, Suvankar Ghorai, Saravana K. K. Mani, Ananta K. Ghosh

**Affiliations:** Department of Biotechnology, Indian Institute of Technology Kharagpur, Kharagpur, 721302 West Bengal India

## Expression of Concern to: Virology Journal 2010; 7:181 http://www.virologyj.com/content/7/1/181

The Editor-in-Chief is issuing an editorial expression of concerns for this article [1]. Concerns were raised regarding a number of figures in this article, specifically:

Figure 6C: the blots in lanes 4 and 5 appear to be very similar

Figure 7: the native virion particle panel for pH-7.3 (B) appears to partially overlap with the VLP containing AMCPV S3 panel for pH-7.3 (B)

Figure 7: the VLP containing AmCPV S3 panel for pH-4 (A) appears to partially overlap with the VLP containing AmCPV S3 panel for pH-12 (C)

Author Ananta K Ghosh has stated the original data are no longer available. Readers are urged to use caution when interpreting the results related to the data presented in these figures.

Ananta K Ghosh agrees to this EEoC. The editor was not able to obtain a current email address for Mrinmay Chakrabarti, Suvankar Ghorai, Saravana KK Mani.

## References

[1] Chakrabarti et al. Virol J. 2010, 7:181 http://www.virologyj.com/content/7/1/181

